# Genetic structure of *Malus sylvestris* and potential link with preference/performance by the rosy apple aphid pest *Dysaphis plantaginea*

**DOI:** 10.1038/s41598-021-85014-x

**Published:** 2021-03-11

**Authors:** Thomas Denoirjean, Géraldine Doury, Amandine Cornille, Xilong Chen, Thierry Hance, Arnaud Ameline

**Affiliations:** 1 UMR CNRS 7058 EDYSAN (Écologie et Dynamique des Systèmes Anthropisés), Université de Picardie Jules Verne, 33 rue St Leu, 80039 Amiens Cedex, France; 2 Université Paris Saclay, INRAE, CNRS, AgroParisTech, GQE - Le Moulon, 91190 Gif-sur-Yvette, France; 3 Earth and Life Institute, Biodiversity Research Centre, UC Louvain, ELIB – Croix du sud 4–5 bte L7.07.04, 1348 Louvain-la-Neuve, Belgium

**Keywords:** Behavioural ecology, Biodiversity, Ecological genetics, Ecophysiology, Plant breeding, Plant genetics, Population genetics, Plant breeding, Plant domestication, Plant genetics, Behavioural ecology, Biodiversity, Ecological genetics, Ecophysiology, Ecology, Genetics, Plant sciences, Ecology, Zoology, Animal behaviour, Animal physiology, Entomology

## Abstract

The European crabapple *Malus sylvestris*, a crop wild relative of *Malus domestica*, is a major contributor to the cultivated apple genome and represents a potential source of interesting alleles or genes, particularly pest resistance traits. An original approach was used to explore the trophic interaction between *M. sylvestris* populations and its pest, the rosy apple aphid (*Dysaphis plantaginea*). Using 13 microsatellite markers, population genetic structure and level of crop-to-wild introgressions were inferred between *M. sylvestris* seedlings from three sites in Europe (Denmark, France, Romania), and *M. domestica* seedlings. Genetically characterized plants were also used to analyze aphid feeding behavior and fitness parameters. First, aphids submitted to two genetically close *M. sylvestris* populations (the Danish and French) exhibited similar behavioral parameters, suggesting similar patterns of resistance in these host plants. Second, the Romanian *M. sylvestris* population was most closely genetically related to *M. domestica*. Although the two plant genetic backgrounds were significantly differentiated, they showed comparable levels of sensitivity to *D. plantaginea* infestation. Third, aphid fitness parameters were not significantly impacted by the host plant’s genetic background. Finally, crop-to-wild introgression seemed to significantly drive resistance to *D. plantaginea* independent of host plant population genetic structure, with hybrids being less suitable hosts.

## Introduction

Intense farming practices can lead to harmful impacts on the environment and human health. It is therefore urgent to promote eco-friendly agricultural management while feeding a growing world population^1^. In this case, new breeding strategies making use of wild untapped genetic diversity could become a promising opportunity to provide farmers with crops less dependent on chemical inputs without heavy drawbacks to productivity^2^. Crop breeding programs often rely on wild species that are either phylogenetically close to a crop and/or that have played a primary role in the crop domestication history. Such plants have been named “crop wild relatives” (or CWR). Although the use of CWR in breeding programs has the potential to fulfill numerous agronomic needs (e.g. yield increase or abiotic stress adaptation), the major focus of CWR research concerns their ability to enhance crop resistance against pathogens and pests^3^.

In the context of breeding programs relying on CWR genetic traits, the cultivated apple tree *Malus domestica* Borkh appears to be an ideal model system for reducing the environmental impacts of food production. Indeed, *M. domestica* is one of the most important fruit tree crops in the world (http://faostat.fao.org/). In Europe, the apple tree is severely attacked by several pests and pathogens, and therefore apple production relies heavily on the use of pesticides. To reduce reliance on pesticide applications, apple breeding programs should test potential sources of resistance alleles such as the three potential local wild apple species in Eurasia. Population genetics analyses revealed that the cultivated *M. domestica* originated from the wild apple *Malus sieversii* Ledeb. in the Tian Shan mountains located in Central Asia^4,5^. From there, the cultivated apple continued its journey along the Silk Routes where it hybridized with local wild apple tree species: first to a little extent with *Malus orientalis* Uglitz. in the Caucasus, and later on (~ 1500 YA), massively in Europe with *Malus sylvestris* (L.) Mill.^4^. *Malus sieversii* is therefore considered the progenitor of the cultivated apple while *M. sylvestris* is considered the second main contributor of apple genetic diversity through recent wild-to-crop introgressions^5^. Through introgression and phylogenetic closeness, *M. sieversii*, *M. orientalis,* and *M. sylvestris* are all considered CWR of cultivated apple. The need to assess their value as future sources for future breeding programs is urgent because these species are currently threatened by local crop-to-wild gene flow^6–9^.

The search for resistance on *M. domestica* wild relatives has been carried out mostly against pathogens, whereas studies describing resistance to pests are less frequent^10^. Among these, the *M. floribunda* Siebold clone 821 proved to be a major source of resistance genes to apple scab *Venturia inaequalis* (Cooke) Winter^11^, fire blight *Erwinia amylovora* Burrill^12^ and the rosy apple aphid (RAA), *Dysaphis plantaginea* Passerini^13^. However, only a handful of studies have investigated resistance to pests and pathogens of CWR involved in the domestication of the apple. One study^14^ compared the levels of resistance against fire blight among 51 different genotypes of *M. sieversii* collected in Kazakhstan and Kyrgyzstan. Similarly the resistance of 194 M*. sieversii* accessions belonging to four distinct genetic groups^15^ was evaluated concurrently with nine different *M. domestica* cultivars^16^. Various fire blight resistance levels were observed among *M. sieversii* genetic clusters with two of them exhibiting the highest resistance levels among tested genotypes. Furthermore, a distinct resistance mechanism was revealed when several wild accessions were compared with cultivated apple cultivars using shoot inoculation in orchards. Specifically, fire blight infection rates were lower for several *M. sieversii* accessions but when successful, infection led to greater damage in these trees. Concerning resistance against insect pests, another study^17^ quantified the resistance of 19 M*. domestica* cultivars and two of its CWR, *M. sylvestris* and *M. kirghisorum*, to the florivorous apple blossom weevil, *Anthonomus pomorum* L.. The authors compared weevil resistance levels between the cultivated apple to the wild species. The species *M. sylvestris* and *M. kirghisorum* Al. Fed. & Fed. appeared to be more sensitive to *A. pomorum* but also supported a more abundant community of the weevil’s natural enemies.

Among the pool of CRW apple species with putative benefits for cultivated apple breeding programs, *M. sylvestris* appears to be largely underexploited. For European apple production, *M. sylvestris* breeding presents several advantages including a shared local environment with *M. domestica*. Previous population genetic analyses of *M sylvestris* populations using microsatellite markers revealed five genetic clusters spread throughout Scandinavia, Western Europe (mostly in France), Eastern Europe, Central Europe, and Italy^6,7^. These five populations may possess adaptive alleles associated with specific environmental conditions or local parasites. However, responses to pathogens and pests among these wild apple genetic groups have yet to be explored.

The rosy apple aphid *D. plantaginea* (RAA) is the major aphid pest of the cultivated apple in Europe, Maghreb, and North America. This aphid species feeds on sap drawn from the phloem and develops at the apex of branches and/or on the most recently developed leaves. In addition to sap extraction, RAA saliva secretion provokes leaf-rolling and impairs shoot growth, greatly reducing yield^18^. Plant defenses against aphids include various strategies disrupting aphid preference, particularly through their host-plant colonization process^19^ that can be characterized by the potential success throughout six behavioral phases: (1) long- and short-range of host-plant perception, (2) plant contact and assessment of surface cues, (3) epidermal probing, (4) stylet pathway activity, (5) phloem penetration and salivation, and (6) phloem acceptance and ingestion. This colonization is finalized by entering a reproductive phase which can be characterized by fecundity and adult survival traits as aphid performance. In this study, a host plant was considered resistant to RAA from the moment it negatively impacted the preference and/or performance of the pest.

The current work explores the RAA behavior/physiology on a crop wild relative of the cultivated apple, taking into account intra-specific CWR population structure and level of crop-to-wild gene flow. This manuscript describes four research objectives: (1) An original sample collection was built from 42 wild apple plants grown from field-collected seeds derived from three of the five known *M. sylvestris* European populations^7^ and 14 cultivated apple plants derived from breeding crosses of *M. domestica* cultivars. (2) The 56 plants sampled were genetically characterized using 13 microsatellite markers^4^. Using population genetics inference, their genetic statuses were determined (i.e. belonging to the Western, Scandinavian and Eastern *M. sylvestris* populations, or to the *M. domestica* gene pool). The degree of crop-to-wild introgression was also assessed among these plants. These genetically characterized plants were then used for behavioral and physiological assays that tested for putative RAA resistance in a CWR of *M. domestica*. (3) For each plant population identified, aphid preference was tested based on feeding behavior measured with the electropenetrography (EPG) technique. (4) As a proxy for measuring fitness, adult fecundity, survival, and adult weight measures were used to determine aphid performance on each of the identified plant populations^20^.

## Materials and methods

### Plant and insect materials

A total of 56 apple plants were grown from seeds and sampled for this study. Cultivated apple plants resulting from crosses between various cultivated apple varieties were used (*M. domestica*, referred to as “Dom”, *N* = 14, Table S1). The seeds were kindly provided by INRAE IRHS Angers that performed every year crosses for apple breeding programs. A total of 42 *M. sylvestris* plants were grown from field-collected seeds. These wild apple seeds originated from three out of the five known European wild apple populations (referred to as Danish: Syl_Dk, French: Syl_Fr and Romanian: Syl_Ro, *N* = 14 per population). Each population was represented by a single sampling site, and within each site, each seed was sampled on a single mother tree, so that each seedling has a different parental origin. Though *M. domestica* is usually grafted, new plants were grown from seed to eliminate the rootstock effect.

After field sampling, seeds were stored at -20 °C before vernalization for the experiment. Seeds were then vernalized for three months at 4 °C in the dark, then grown in controlled conditions for two months before being individually transferred to 3 L pots containing commercial sterilized potting soil. Potted plants were grown in a growth chamber for four weeks under the following conditions: 20 ± 1 °C, 75 ± 5% Relative Humidity (RH), and a 16:8 light:dark (L:D) photoperiod. The 56 plants were then genotyped using 13 previously published microsatellite markers (see below) to confirm their genetic status (i.e., belonging to one of the *M. sylvestris* European populations or crop-to-wild/wild-to-wild hybrid).

A single colony of *D. plantaginea* (Hemiptera: Aphididae) was used and provided by INRAE which were sampled as a population in spring 2018 from an apple tree at the Agrocampus Ouest orchard (Angers, France) (Philippe Robert, personal communication). This aphid population was mass reared without differentiating individual aphid clones on *M. domestica* cv. “Jonagold” plants obtained by in vitro multiplication^21^. Pots containing three plants (90 × 90 × 70 mm) were placed in a Plexiglas cube (50 cm). Mass rearing and experiments were performed in growth chambers under 20 ± 1 °C, 60 ± 5% RH, and a 16:8 L:D cycle.

Synchronized first instar nymphs were obtained by placing parthenogenetic adult females on plantlets for 24 h before removing them. They were then reared on *M. domestica* cv. “Jonagold” plants inside Plexiglas aerated boxes (36 × 24 × 14 cm) for ten days then used as the young adult RAA for the behavioral/performance experiments.

### Apple population genetic diversity and structure

Genomic DNA was extracted with the NucleoSpin plant DNA extraction kit II (Macherey & Nagel, Düren, Germany) according to the manufacturer’s instructions. Microsatellites were amplified by multiplex PCR, with the Multiplex PCR Kit (QIAGEN, Inc.). We used 13 microsatellite markers, Ch01f02, Ch01f03, Ch01h01, Ch01h10, Ch02c06, Ch02c09, Ch02c11, Ch02d08, Ch03d07, Ch04c07, Ch05f06, GD12, and Hi02c07 in four multiplexes (MP01, MP02, MP03, MP04)^4^. PCR were performed in a final reaction volume of 15 ml (7.5 ml of QIAGEN Multiplex Master Mix, 10–20 mM of each primer, with the forward primer labelled with a fluorescent dye and 10 ng of template DNA) (See^4^ for more details). The final volume was achieved with distilled water. A touch-down PCR program (initial annealing temperature of 60 °C, decreasing by 1 °C per cycle down to 55 °C) was used. Genotyping was performed on the GENTYANE platform (INRAE Clermont-Ferrand) using an ABI PRISM X3730XL, with 2 ml of GS500LIZ size standard (Applied Biosystems). Alleles were scored with GENEMAPPER 4.0 software (Applied Biosystems). Only multilocus genotypes with < 10% missing data were retained.

The genetic status of each seedling was assessed using the individual-based Bayesian clustering method implemented in STRUCTURE 2.3.3^22^. STRUCTURE makes use of Markov Chain Monte Carlo (MCMC) simulations to infer the proportion of ancestry of genotypes from *K* distinct clusters. The underlying algorithm attempts to minimize deviations from Hardy–Weinberg and linkage disequilibria. STRUCTURE was run from *K* = 1 to *K* = 8, ten independent runs were carried out for each *K* and 500,000 MCMC iterations were used after a burn-in of 50,000 steps. CLUMPAK (Greedy algorithm)^23^ was used to look for distinct modes among the 10 replicated runs of each *K*. STRUCTURE analyses were run for the full dataset (*N* = 55, DNA could not be extracted from one Romanian seedling), and included as well 40 *M. domestica* genotypes as a reference for the cultivated apple gene pool^6^. We determined the strongest level of genetic structure using Δ*K*^24^, as implemented in the online post processing software Structure Harvester^25^. However, the *K* identified by this criterion often does not correspond to the finest biologically relevant population structure^6,7,26,27^. A lack of consideration of intraspecies genetic structure in STRUCTURE analyses can bias the interpretation of introgression rates. We therefore visualized the bar plots and chose the *K* value for which all clusters had well assigned individuals while no further well-delimited and biogeographically relevant clusters could be identified for higher *K* values.

Once the best *K* chosen, wild plants assigned to the cultivated gene pool with a membership coefficient > 0.1 were classified as crop-to-wild hybrids (i.e., introgressed by *M. domestica*). Once crop-wild hybrids removed, plants assigned to a given wild gene pool with a cumulated membership coefficient > 0.9 were defined as “pure wild” individuals. Plants assigned to the wild gene pool with a cumulated membership coefficient < 0.9 to a given wild apple gene pool were defined as wild-wild-hybrids The pure, crop-to-wild and wild-wild hybrids were included as factors in the statistical analyses. Pure seedlings were then assigned to a population (i.e., group of plants with a cumulated membership coefficient of up to 0.90 for a given wild apple cluster). Pure populations from the same geographic origin (i.e., Romania or France or Denmark) which showed (1) weak genetic differentiation with other wild populations (2) low number of individuals were merged. The “population” was then used as a factor for statistical analyses on physiological and behavioral assays. Population genetics statistics were estimated with Genodive^28^ for each “pure” wild apple population including expected and observed heterozygosities, Weir and Cockerham F-statistics, Jost’s D, and deviations from Hardy–Weinberg equilibrium.

### *Dysaphis plantaginea* feeding behavior

The feeding behavior of the RAA was investigated using the electrical penetration graph (EPG) method^29^. Individual aphids were connected to the Giga-8 DC-EPG amplifier, each being placed on the abaxial side of a new growing leaf of an individual plant. The recordings were performed continuously for 8 h during the photophase inside a Faraday cage. Acquisition and analysis of the EPG waveforms were carried out using the PROBE 3.5 software (EPG Systems, www.epgsystems.eu). Parameters from the recorded waveforms were calculated with the EPG-Calc 6.1.7 software^30^. They were based on different EPG waveforms^31^ corresponding to: (Pr) stylet activity within plant tissues; (C) stylet pathways in plant tissues except phloem and xylem; (E1) salivation in phloem elements; (E2) passive phloem sap ingestion; (G) active xylem sap ingestion; and (F) derailed stylet mechanics. A total of eight plants per *M. sylvestris* population (Syl_Dk, Syl_Fr, Syl_Ro) or *M. domestica* (Dom) genetic group were used for the EPG measurements. EPG records were obtained from 25 aphids for *M. domestica*, and from 27 aphids for each *M. sylvestris* population.

### *Dysaphis plantaginea* performance

Two to three clip-cages were installed on 12 plants per genetic group identified in this study. Each cage contained an individual, synchronized aphid and was enclosed on a newly grown leaf. For each synchronized adult, observations were assessed every 24 h for 10 days. Survival (i.e., the duration of adult survival over the period of 10 days) and daily fecundity (i.e., the number of newly larviposited nymphs) were collected for 25 adults for *M. domestica*, and 28 to 29 adults for each of the three *M. sylvestris* populations.

To measure aphid weight, newly larviposited nymphs were enclosed for nine days in clip-cages on newly grown leaves similar to the above. For each plant genetic background, up to 20 aphids (i.e. young adults that had not larviposited yet) were then collected and stored in a freezer at – 80 °C. Each individual aphid was weighed using an electronic precision balance (Mettler M3, class 1, Max: 3 g Low: 1 µg, T =  − 3G [dd] = 1 µg).

### Statistical analyses

All statistical analyses were performed using the R software version 3.6.2 (The R Foundation, https://www.r-project.org/). Generalized linear models (GLM) with a likelihood ratio and Chi-square test were used to assess whether there was an effect of the host plant on aphid feeding behavior and performance. The apple tree genotype was included as the main factor. Data on daily aphid fecundity and some EPG parameters describing the number of occurrences of a particular phase (e.g. “n_E2”) were not normally distributed (count data), accordingly a GLM was carried out using respectively a quasi-Poisson and a Poisson distribution; a quasi-likelihood function was used to correct for over-dispersion, and Log was specified as the link function in the model. EPG data on feeding phase durations (e.g. duration of phloem sap ingestion “s_E2”) and aphid weight were not normally distributed, so a GLM using a Gamma (link = “inverse”) distribution was carried out. Analysis of the time before the first probe (“t.1Pr”) and before the first phloem sap ingestion (“t.1E2”) and adult survival has been carried out using the Cox proportional hazards (CPH) regression model, which is adapted to treat time-dependent parameters. Absence of an EPG reading were treated as missing values. The assumption of validity of proportional hazards was validated using the function “coxph” (package R: “survival”, version 3.1.8: https://github.com/therneau/survival). To assess whether the crop-to-wild hybrid status had a plant-mediated effect on RAA feeding behavior and performance, the same statistical tests were carried out with the hybrid statuts (i.e. “wild pure” or “wild-crop hybrid”) as the fixed factor while restricting the data set to the *M. sylvestris* populations only.

Finally, because of their close genetic relatedness, the Danish and French wild apples plants (Syl_Dk and Syl_Fr, respectively) were grouped together, as well as the *M. domestica* and the Romanian wild apple plants (Dom and Syl_Ro, respectively). A Monte-Carlo permutation test (999 replicates) was conducted to test for the significance of the differences of median of EPG phases duration, daily fecundity and weight between aphids submitted to these two host groups. Analysis of the time before the first probe (“t.1Pr”) and before the first phloem sap ingestion (“t.1E2”) and adult survival has been carried out using the CPH regression model. The function “randtest” (package R: “ade4”: https://cran.r-project.org/web/packages/ade4/ade4.pdf) was run to access the significance of the observed differences.

The fit of all GLM was controlled by a visual evaluation of residuals and QQ plots. Concerning QQ plots, the distribution of the series were considered to follow the chosen theoretical law if the points of the graph were roughly aligned on a straight line. Any other structuring of the points (curvature(s), many distant points, etc.) indicated the opposite. GLM post-hoc comparisons were carried out by pairwise comparisons using estimated marginal means (package R: “emmeans”, https://cran.r-project.org/web/packages/emmeans/emmeans.pdf).

### Ethics approval

The article does not contain any studies with human participants or vertebrate animals.

## Results

### Population structure and detection of crop-wild and wild-wild hybrids

**Figure 1:**
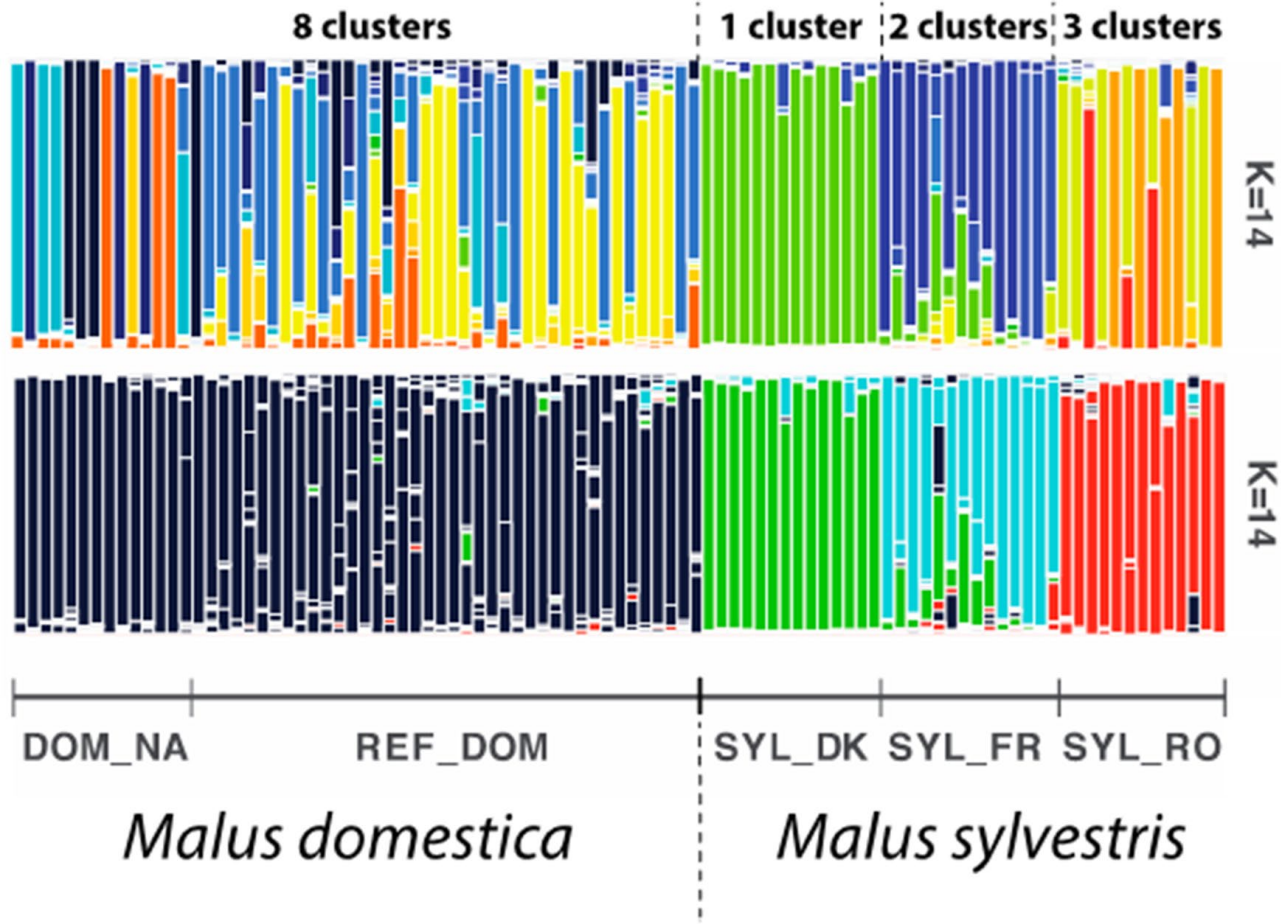
Assessment of the genetic status of the wild and cultivated apple seedlings (*Malus sylvestris* and *Malus domestica*, respectively) used in this study. Upper barplot: Population structure inferred with STRUCTURE for *K* = 14 for the Romanian, French, Danish *M. sylvestris* seedlings, and the *M. domestica* seedlings. STRUCTURE detected eight cultivated gene pools, including the 40 M*. domestica* reference cultivars (DOM_REF, 40 reference *M. domestica*) and the cultivated apple seedling used in this study (DOM_NA, *N* = 14). The Romanian seedlings (SYL_RO, *N* = 13) clustered into three clusters (orange, red and yellow colors), the French seedlings (SYL_FR, *N* = 14) into two clusters (light and dark blue color), and the Danish seedlings (SYL_DK, *N* = 14) into one cluster (green color). Lower barplot: For the sake of visualization the eight cultivated gene pools were coloured in dark blue (lower barplot), the three Romanian clusters in red, the two French clusters in light blue, and the Danish stayed light green.

STRUCTURE analyses revealed a clear split between *M. domestica* and *M. sylvestris* seedlings for *K* = 3 (Supplementary Fig. S1). However, failing to take the population structure of the wild species into account can lead to spurious signals of introgression from crop species^26^. We therefore analyzed the structure of *M. sylvestris* and identified that the Romanian, French, and Danish *M. sylvestris* seedlings formed distinct genetic clusters from each other for *K* = 14. For *K* = 14 the *M. domestica* seedlings used in the experiment grouped with the 40 reference *M. domestica* (Fig. 1 and Supplementary Fig. S1). The use of *K* values > 14 uncovered no further structure within *M. sylvestris*, indicating *K* = 14 captured > 99% of the genetic variance. STRUCTURE analysis detected eight clusters for *M. domestica* and six clusters for *M. sylvestris*. For *K* = 14, STRUCTURE revealed a clear partition between four discrete groups (Fig. 1 and Supplementary Fig. S1): (1) *M. domestica*, including *M. domestica* seedlings and the 40 reference samples, divided in eight admixed genetic groups, (2) the Romanian seedlings, divided into three genetic groups (orange, red, yellow), (3) the French seedlings divided into two genetic groups (dark and light blue, respectively), and (4) the Danish seedlings formed a single distinct genetic group (green). Note that the French and Danish samples only split from *K* = 11. This weak genetic structure was further validated by the relatively low F_ST_ and Jost’s D values among those two groups (F_ST_ = 0.08, *P* < 0.01, Table S2). We therefore used cumulative membership coefficient of each seedling in the six *M. sylvestris* or the eight *M. domestica* genetic groups in subsequent analyses to identify crop-to-wild and wild-wild hybrid genotypes.

For the 41 seedlings identified *a priori* as *M. sylvestris*, four genotypes (three French seedlings and one Romanian seedling, 9.7% of the *M. sylvestris* seedlings) showed signs of introgression from *M. domestica* (i.e., cumulative membership coefficients > 0.1 into the cumulated *M. domestica* gene pool, Supplementary Table S1). A total of four wild-wild hybrids were detected (i.e., individuals with cumulative membership coefficients into the three French genepools < 0.9, Supplementary Table S1), including three French-Danish, and one Romanian-French hybrids. Genetic diversity estimates for each population, and genetic differentiation estimates among populations (excluding crop-wild and wild-wild hybrids) are provided in Tables S1 and S2. Note that, once the hybrids were removed, the Romanian wild apple population (FR_RO) was the genetically closest wild apple population to the cultivated apple (i.e. DOM_REF and DOM_NA, F_ST(REF_DOM-SYL_RO)_ = 0.09 and F_ST(REF_NA-SYL_RO)_ = 0.11, respectively, *P* < 0.001), and the Danish and French wild apple populations were still the most genetically closely related, followed by the Romanian and the French wild apple populations (Supplementary Table S2).

### Effect of the host apple species and population on *Dysaphis plantaginea* feeding behavior

Feeding phases and associated analytical results are described in Table 1 for aphids reared on *M. domestica* and trees of each *M. sylvestris* population. Concerning general probing (Pr, parameters 1–3), the total duration of stylet activity in the plants (Pr, parameter 2) lasted on average about 6 h (out of 8 recorded hours) and was not significantly different among aphids, whatever the genetic background of their host plant (GLM using Gamma distribution: χ^2^ = 0.60, Df = 3, *P* = 0.60). This global activity was composed of an average of 13 probing events (Pr, parameter 3), again without any significant difference between aphids on the host plants with different genetic background (GLM using Poisson distribution: χ^2^ = 0.14, Df = 3, *P* = 0.14). This is despite the fact that the mean number of probes tended to be smaller on *M. domestica* host plants. Similarly, there was no significant difference between the time to first probe (Pr, parameter 1) (CPH: χ^2^ = 1.49, Df = 3, *P* = 0.68). The pathway phase (C, parameters 4 and 5) comprised on average of 60% of the total activity. There was no significant difference for its total duration (C, parameter 4) (GLM using Gamma distribution: χ^2^ = 3.13, Df = 3, *P* = 0.37) or for the number of occurrence (C, parameter 5) (GLM using Poisson distribution: χ^2^ = 5.49, Df = 3, *P* = 0.14).

**Table 1:** Feeding phases (mean ± standard error of the mean) of *Dysaphis plantaginea* feeding on plants belonging to the three *Malus sylvestris* populations (i.e., Danish, French and Romanian, hereafter referred to as “Syl_Dk”, “Syl_Fr”, “Syl_Ro”, respectively) and to the *Malus domestica* genepool (“Dom”).

EPG classes	GLM/Cox models *P* value	Dom	Syl_Dk	Syl_Fr	Syl_Ro
General probing behavior and pathway phase		*(n* = *25)*	*(n* = *27)*	*(n* = *27)*	*(n* = *27)*
1. Time to first probe (min)	0.68 (NS)	20.67 ± 6.52	22.77 ± 7.00	14.67 ± 2.56	25.88 ± 6.12
2. Total duration of probing (Pr) (min)	0.60 (NS)	374.66 ± 12.55	361.50 ± 10.83	377.63 ± 11.31	359.90 ± 11.13
3. Number of probes (Pr)	0.14 (NS)	11.44 ± 1.26	12.96 ± 1.98	12.78 ± 1.50	13.70 ± 1.37
4. Total duration of pathway phase (C) (min)	0.37 (NS)	218.08 ± 17.05	250.30 ± 11.53	228.86 ± 12.47	217.33 ± 16.81
5. Number of pathway phases (C)	0.14 (NS)	16.32 ± 1.42	18.96 ± 1.98	17.56 ± 1.40	18.19 ± 1.32
Phloem phase		*(n* = *21)*	*(n* = *22)*	*(n* = *20)*	*(n* = *20)*
6. Total duration of phloem salivation (E1) (min)	< 0.001(***)	9.09 ± 1.62 a	3.04 ± 0.59 b	2.58 ± 0.46 b	8.28 ± 1.62 a
7. Number of phloem salivation (E1)	< 0.001(***)	4.80 ± 0.76 a	3.22 ± 0.53 bc	2.52 ± 0.51 c	4.07 ± 0.68 ab
		*(n* = *19)*	*(n* = *20)*	*(n* = *18)*	*(n* = *19)*
8. Time to first phloem ingestion (E2) (min)	0.48 (NS)	174.38 ± 25.13	230.56 ± 28.55	253.90 ± 30.16	183.95 ± 20.93
9. Total duration of phloem sap ingestion (E2) (min)	0.008 (**)	91.39 ± 18.68 a	32.25 ± 7.44 b	71.15 ± 19.19ab	86.75 ± 18.04 a
10. Number of phloem ingestion (E2)	0.006 (**)	3.24 ± 0.57 a	2.37 ± 0.42 ab	1.96 ± 0.38 b	3.19 ± 0.57 a
Other phases		*(n* = *5)*	*(n* = *0)*	*(n* = *2)*	*(n* = *2)*
11. Total duration of xylem ingestion (G) (min)	–	134.85 ± 52.83	–	50.04 ± 24.70	39.07 ± 12.62
		*(n* = *13)*	*(n* = *14)*	*(n* = *10)*	*(n* = *12)*
12. Total duration of stylet derailment (F) (min)	0.59 (NS)	82.76 ± 13.23	93.28 ± 10.06	108.66 ± 19.94	109.42 ± 23.26

The letters within a row indicate significant differences associated with pairwise comparisons using estimated marginal means.

*P < 0.05; **P < 0.01; ***P < 0.001 associated with GLM models (using respectively Poisson and Gamma distribution for the number and total duration of feeding phases) or Cox models (for “time to first phase”) (degree of freedom = 3 for each test).

Concerning the phloemian phase (parameters 6–10), most parameters revealed differences among aphids fed on trees belonging to the three wild apple populations and the cultivated gene pool. The mean duration of salivation within phloem was significantly shorter for aphids submitted to French and Danish *M. sylvestris* plants compared with aphids submitted to *M. domestica* and Romanian *M. sylvestris* plants (E1, parameter 6) (GLM using Gamma distribution: χ^2^ = 35.02, Df = 3, *P* < 0.001). Aphids fed on Danish and French *M. sylvestris* plants salivated three times less compared to aphids fed on *M. domestica* and Romanian *M. sylvestris* plants. Aphids submitted to French and Danish *M. sylvestris* plants displayed a smaller number of salivations within phloem compared to aphids on *M. domestica* whereas aphids submitted to French *M. sylvestris* plants had a significantly shorter number of salivations within phloem compared to those on Romanian *M. sylvestris* plants (E1, parameter 7) (GLM using Poisson distribution: χ^2^ = 21.55, Df = 3, *P* < 0.001). The mean duration of phloem ingestion (E2, parameter 9) was significantly shorter for aphids on the Danish *M. sylvestris* compared to aphids on *M. domestica* and the Romanian *M. sylvestris* plants (GLM using Gamma distribution: χ^2^ = 12.50, Df = 3, *P* = 0.08). The mean proportion of the time dedicated to phloem ingestion (E2) was variable depending on the host plant genetic background: from 9% (Syl_Dk) to 24% (Syl_Ro/Dom) within the general probing activity. The number of phloem ingestion (E2, parameter 10) was significantly smaller for aphids submitted to the French *M. sylvestris* compared to those submitted to *M. domestica* and the Romanian *M. sylvestris* plants (GLM using Poisson distribution: χ^2^ = 11.89, Df = 3, *P* = 0.06).

Finally, the duration of the time needed by an aphid to reach the phloem (E2, parameter 8) tended to be shorter on *M. domestica* or Romanian *M. sylvestris* plants (around 3 h) than on Danish and French *M. sylvestris* plants (around 4 h), though no significant difference was observed (CPH: χ^2^ = 2.48, Df = 3, *P* = 0.48). Considering xylem ingestion (G, parameter 11) aphids submitted to Danish *M. sylvestris* plants did not ingest raw sap, whereas a few aphids ingested xylem on the French and Romanian *M. sylvestris* plants. Altogether, not enough aphids displayed this behavior to conduct statistical analysis. Almost half of the aphids presented stylet derailment (F, parameter 12) for an average total duration of roughly 1.5 h, that was not statistically different between the different host plant genetic backgrounds (GLM using Gamma distribution: χ^2^ = 0.59, Df = 3, *P* = 0.59).

Considering the genetic proximity of the Danish and French *M. sylvestris* populations (Table S2), as well as the genetic proximity of the Romanian *M. sylvestris* and *M. domestica*, the two-by-two pairing of datasets (i.e., Syl_Dk/Syl_Fr vs Dom/Syl_Ro) revealed that the total duration of phloem salivation (Monte-Carlo permutation test, *P* = 0.001) and the total duration of phloem sap ingestion (Monte-Carlo permutation test, *P* = 0.016) were significantly longer for the Dom/Syl_Ro pair. Regardless *M. sylvestris* populations, the duration of phloem sap ingestion phase was significantly affected by the host plant hybrid status and was shorter for hybrids compared to pure *M. sylvestris* (GLM using Gamma distribution: χ^2^ = 4.23, Df = 1, *P* = 0.04).

### Effect of the host plant population on *Dysaphis plantaginea* fitness parameters

The impact of host plant genetic background on RAA fitness and associated statistical analyses are presented in Table 2. There was no significant difference for daily fecundities (GLM using quasi-Poisson distribution: χ^2^ = 1.49, Df = 3, *P* > 0.05) between aphids raised on the four plant genetic backgrounds. Similarly, there was no significant difference for survival (CPH: χ^2^ = 3.39, Df = 3, *P* > 0.05). Aphid weight was significantly impacted by the plant genetic background: the weight of aphids raised on *M. domestica* was significantly greater (GLM using Gamma distribution: χ^2^ = 8.10, Df = 3, *P* < 0.05) than that of aphids raised on the Danish *M. sylvestris* plants. Again taking into consideration the genetic proximity between the Danish and French *M. sylvestris* as well as the Romanian *M. sylvestris* and *M. domestica*, the two-by-two pairing of datasets (Syl_Dk/Syl_Fr vs Dom/Syl_Ro) revealed no significant difference for all fitness parameters. Regardless *M. sylvestris* population, aphid weight was significantly affected by the host-plant hybrid status: aphids raised on hybrids displayed smaller weights compared to those on pure *M. sylvestris* (GLM using Gamma distribution: χ^2^ = 5.16, Df = 1, *P* = 0.02), whereas neither fecundity nor survival were impacted.

**Table 2:** Fitness parameters (mean ± standard error of the mean) for *Dysaphis plantaginea* reared on plants belonging to three *Malus sylvestris* populations (Danish, French and Romanian, i.e. hereafter referred as to “Syl_Dk”, “Syl_Fr”, “Syl_Ro”, respectively) and to the *Malus domestica* genepool (“Dom”).

Parameters	GLM/Cox models *P* value	Dom	Syl_Dk	Syl_Fr	Syl_Ro
		*n* = *25*	*n* = *28*	*n* = *29*	*n* = *28*
Daily fecundity	0.69 (NS)	2.33 ± 0.26	2.46 ± 0.26	2.38 ± 0.22	2.08 ± 0.22
Survival (days)	0.34 (NS)	9.20 ± 0.34	9.75 ± 0.14	9.28 ± 0.39	9.50 ± 0.31
		*n* = *56*	*n* = *73*	*n* = *80*	*n* = *80*
Aphid weight (µg)	0.043 (*)	557.88 ± 39.05 a	427.92 ± 28.54 b	476.30 ± 22.84 ab	490.26 ± 32.20 ab

The letters within a row indicate significant differences associated with pairwise comparisons using estimated marginal means.

*P < 0.05; **P < 0.01; ***P < 0.001 associated with GLM models (using respectively a quasi-Poisson for daily fecundity and Gamma distribution aphid weight) or Cox models (for survival) (degree of freedom = 3 for each test).

## Discussion

This is the first study reporting differences in phytophagous pest preference that are congruent with the genetic relationship between a wild relative and a cultivated plant with which it has introgressed. Population genetic analyses also revealed weak genetic differentiation between the Danish and French *M. sylvestris* wild apple populations. Accordingly, behavioral assays of aphids submitted to plants from these two populations showed similar patterns suggesting antixenosis resistance^32^. Likewise, the Romanian *M. sylvestris* host population was strongly differentiated from *M. domestica* but was also the most closely genetically related to the wild apple population. Consistent with this observation, the Romanian wild apple population and *M. domestica* showed comparable levels of sensitivity to RAA. Crop-to-wild introgression appeared to drive resistance to RAA independent of population genetic structure.

This study revealed a putative link between aphid preference and the genetic structure among wild and cultivated apple populations. The population structure inferred here stingingly matched the one previously observed for the European wild apple^7,33^, with five main populations in Europe of which, an Eastern, a French and a Scandinavian. We showed that the French and Danish populations were the genetically closest and sharing the highest number of wild-wild hybrids. Accordingly, we can note that aphids showed similar patterns of feeding behavior and performance when submitted to the two most closely related wild apple populations (the Danish and French populations). When aphids were subjected to the Romanian wild apple population and the cultivated apple, similar patterns of aphid behavior and performance were recorded and were associated with higher preference for the Romanian host-plant than aphids submitted to the Danish and French populations. Aphid behavior was actually congruent with the level of genetic differentiation between the Romanian wild apple and the cultivated apple. Once the recent crop-to-wild hybrids were removed, the Romanian population appeared to be the closest wild apple relative to cultivated *M. domestica*. Genetic proximity of populations is known to drive patterns of resistance against pathogens in the wild apple *M. sieversii*^15,16^. Previous studies revealed variable resistance against pests and pathogens among *M. domestica* CWR^14,16^. In this study, the genetic differentiation between the two paired groups (Syl_Dk/Syl_Fr vs Dom/Syl_Ro) might be associated with a phenotypic differentiation associated with the differences observed in terms of RAA feeding behavior. The genetic proximity of the French/Danish and Romanian/cultivated apple may reflect common evolutionary history, however further investigations are required concerning the evolutionary history of the cultivated apple in Europe. In particular, the relative contributions of each wild apple population, especially the Romanian, to the cultivated apple gene pool remains unknown. Addressing this issue would require much larger sampling among European apple seeds.

Not considering genetic proximity among populations, but only population structure, behavioral analyses with EPG demonstrated a generalized activity for the rosy apple aphid, which did not depend on the genetic background of the host plant. Our results showed that whatever the plant genetic background, the time to first probe was not delayed, meaning that the possible influence of epidermal barriers and/or putative plant volatile organic compound (VOC) repulsive effects could be excluded; VOC on leaf surface could indeed impact aphids behavior^19^. A delayed aphid stylet activity is considered to be due to epidermic factors, as the second phase of host selection involves the assessment of plant surface cues by the aphid. Features such as a thick cuticle or the presence of trichomes are physical parameters that may play a role in aphid resistance^34^. Stylet derailment was displayed on every plant genetic background in the same range of mean duration and the pathway phase was not influenced by the plant genetic background. This means that putative mild physicochemical resistance is present of mesophyll tissues in both wild and cultivated apple. In contrast with the above, significant differences were observed between the four plant genetic backgrounds in phloem-related behavior. The phloemian activity was significantly reduced in terms of the duration of both salivation and ingestion for aphids submitted to the Danish and French *M. sylvestris* populations. Since the average time to reach the phloem was not significantly different between the four plant genetic backgrounds, these differences did not appear to be linked to physical characteristics but due to the phloem chemical composition. Comparison of ascorbic acid glycoside (AAG) content in *M. domestica*, *M. sylvestris* and *M. sieversii* apple fruits revealed that accessions of *M. sylvestris* were distinguished by higher concentrations of AAG^35^. A difference in terms of phenolic compounds among *M. sylvestris* populations could be a possible factor explaining the contrasted phloemian activities observed. In fact, phenolic profile of various *M. domestica* cultivars apple fruits can be linked to field RAA resistance^36^. Further studies involving choice assays towards the four genetic backgrounds should provide a better understanding of RAA preference, and especially of long and short range host-plant perception. Despite the differences recorded in the feeding behavior, no differences were observed in two of the three RAA fitness parameters (survival and fecundity) regardless of host-plant genetic background. As high proportions of aphids could initiate reproduction before accessing the phloem^37^, the results concerning fecundity may be consistent with the absence of significant differences in pathway phase parameters. Aphid biomass assays revealed that only aphids submitted to the cultivated apple tree had greater weights than those submitted to the Danish *M. sylvestris.* This is consistent with the fact that the shortest sap ingestion was observed for aphids submitted to the Danish *M. sylvestris*, whereas aphids reared on *M. domestica* exhibited the longest sap ingestion. Sap ingestion is known to be positively correlated with growth^38^, thus the contrasted preference of RAA was not reflected in RAA performance, except for adult weight.

For the first time, our study also shed light on the impact of domestic introgression in *M. sylvestris* on RAA preference and performance. Previous studies already detected substantial crop-to-wild gene flow in apple trees in Europe^7,8^. Here, we confirmed the occurrence of ongoing crop-to-wild gene flow for the European wild apple. We detected 11% of crop-to-wild hybrids in our dataset, which is half less than previous estimates (23%)^7^. Several reasons can explain this discrepancy, including the narrow spatial geographic area investigated here (four locations versus 62 locations previously) and the lower number of samples used (here 42 vs 1889 M*. sylvestris* trees previously). Note however that the aim of our study was not to investigate the large-scale crop-wild gene flow in *M. sylvestris* but assessing the genetic status of the seedlings used for aphid physiological and behavioral assays to control this effect in the statistical models. Yet, in comparison to previous studies, the detection of crop-wild hybrids in seeds collected in 2016–2017 are proof that recent ongoing crop-to-wild gene flows are still at work in apples, especially in the French populations. Indeed, previous studies rather investigated historical crop-to-wild gene flow as they did not sample seeds but much older mother trees. It is also interesting to note that we detected a clear effect of the hybrid status on RAA preference, in terms of phloem sap ingestion, and on RAA performance, in terms of aphid weight. Our bioassays on aphids revealed that pure *M. sylvestris* were more suitable hosts to RAA than crop-to-wild hybrids. A previous study revealed that crop-to-wild hybrids showed higher plant growth and pollination rates compared to pure wild apples^8^. Here our results would suggest that higher fitness of crop-to-wild hybrids is not only expressed for early developmental traits but is also associated with higher resistance abilities to RAA attacks. However, this question would require further investigation.

Finally, it is worth questioning to what extent *Malus sylvestris* could represent a putative genetic source of resistance for *Malus domestica* breeding programs. The *M. domestica* genetic group studied here could be considered as susceptible when compared with the resistance/susceptibility of *M. domestica* to RAA demonstrated by a previous study also using EPG^39^. Regarding general probing activity and phloemian phases, the mean values of electropenetrography parameters obtained here for aphids submitted to the *M. domestica* genetic group appeared to be close to values obtained on a susceptible cultivar (*M. domestica* cv. Golden Delicious) in comparison to a resistant one (*M. domestica* cv. Florina)^39^. The latter has been identified as strongly resistant to the RAA in numerous studies^13,40–42^. In our study, we observed a gradient of resistance to the RAA for the European wild apple, but less marked than for *M. domestica*. Among *M. domestica* cultivars, resistant cultivars impacted RAA preference through both epidermic and phloemian factors^39^. Indeed, compared to that of aphids on susceptible controls, the feeding behavior of aphids on the resistant Florina cultivar revealed shorter durations for general probing (Pr), phloem salivation phase (E1), xylem sap ingestion (G). Also, none of the aphids ingested phloem sap (i.e. the E2 phase was null) and a significantly longer total duration of stylets derailment was observed when submitted to this resistant cultivar. In our case, *M. sylvestris* did only impact RAA preference through the phloemian phase, with most individuals able to ingest sap, although lasting for short duration. Therefore, RAA preference in the European wild apple studied here only involved one factor, the phloemian phase, in contrast to what was previously observed among *M. domestica* cultivars. Besides, the two-factor aphid response on the *M. domestica* host is also associated with lower RAA performance^13,40–42^. Strikingly, our study showed that only the Danish *M. sylvestris* population negatively impacted both RAA preference and performance compared to *M. domestica*. Thus, the Danish *M. sylvestris* population may be more resistant against RAA than *M. domestica* and would appear to represent a potential source of resistance for *M. domestica* breeding programs, although this CRW candidate did not impact RAA fecundity or survival. However, our results are consistent with previous investigations of pests and pathogens resistance in *Malus domestica* CWR which were involved in its domestication, in which some wild accessions were as sensitive as the cultivated ones^16,17^. Resistance against the pest of CWR would be mainly indirect, as they support greater communities of natural enemies^17^. Thus, as we did not proceed to field validation of our results, we may have overlooked some components of CWR resistance. Investigating RAA performance for several aphid generations would also be worth carrying out on the Danish *M. sylvestris* population, as this host negatively impacted aphid behavior and fitness in terms of weight and these negative effects may have a greater impact over generations. A greater diversity of new resistance genes or alleles against RAA may be present in CWR gene pool involved in the apple domestication. CWR are however largely neglected when it comes to studying their resistance to RAA. To better understand resistance differences at an interspecific level, more CWR species have to be included in experiments, such as *M. orientalis* and *M. sieversii*. However, this would involve exposing RAA to apple CWRs that are absent in its natural environment. Future studies may also investigate cross infestations of aphid populations from different parts of Europe onto CWR populations to truly test for RAA local adaptation. To test for the influence of maternal priming, it would also be interesting to compare relative preferences among the aphids reared on *M. domestica* to the relative preference among aphids reared on *M. sylvestris*.

To conclude, this work tested for the first time preferences and survival of a main pest of apple trees, among genetically distinct groups including wild and cultivated host plants. Identification of resistance adaptations among wild genotypes may help design strategies to improve *M. domestica* plant productivity. But above all, the present study reveals that the search for resistant CWR must not only be based on a genetic structure of wild populations but also on the crop-to-wild gene flow that appears to substantially drive resistance to RAA. In that sense French wild apples, which showed high level of crop-to-wild gene flow, may be good candidates for future breeding programs.

## Supplementary Information

Supplementary Information 1.
