# Supplementary material for: Genetic structure of Malus sylvestris and potential link with preference/performance by the rosy apple aphid pest Dysaphis plantaginea
Source: Sci Rep. 2021 Mar 11;11:5732. doi: 10.1038/s41598-021-85014-x (PMC7970975; doi:10.1038/s41598-021-85014-x)
Supplement: Supplementary file 1 — Supplementary Information 1. [file 41598_2021_85014_MOESM1_ESM.pdf]

Supplementary Information

Genetic structure of *Malus sylvestris* and potential link with preference/performance by the rosy apple aphid pest *Dysaphis plantaginea*

Denoirjean Thomas[1], Doury Géraldine[1], Cornille Amandine[2], Chen Xilong[2], Hance Thierry[3], Ameline Arnaud[1*].

1. UMR CNRS 7058 EDYSAN (Écologie et Dynamique des Systèmes Anthropisés), Université de Picardie Jules Verne, 33 rue St Leu, F-80039 Amiens Cedex, France.

2. GQE– Le Moulon, INRAE, Univ. Paris-Sud, CNRS, AgroParisTech, Université Paris-Saclay, 91190, Gif-sur-Yvette, France.

3. Earth and Life Institute, Biodiversity Research Centre, UC Louvain, ELIB – Croix du sud 4–5 bte L7.07.04, 1348 Louvain-la-Neuve, Belgium.

1. Supplementary figure related to the population structure of *Malus domestica* and Danish, French and Romanian *Malus sylvestris* seedlings

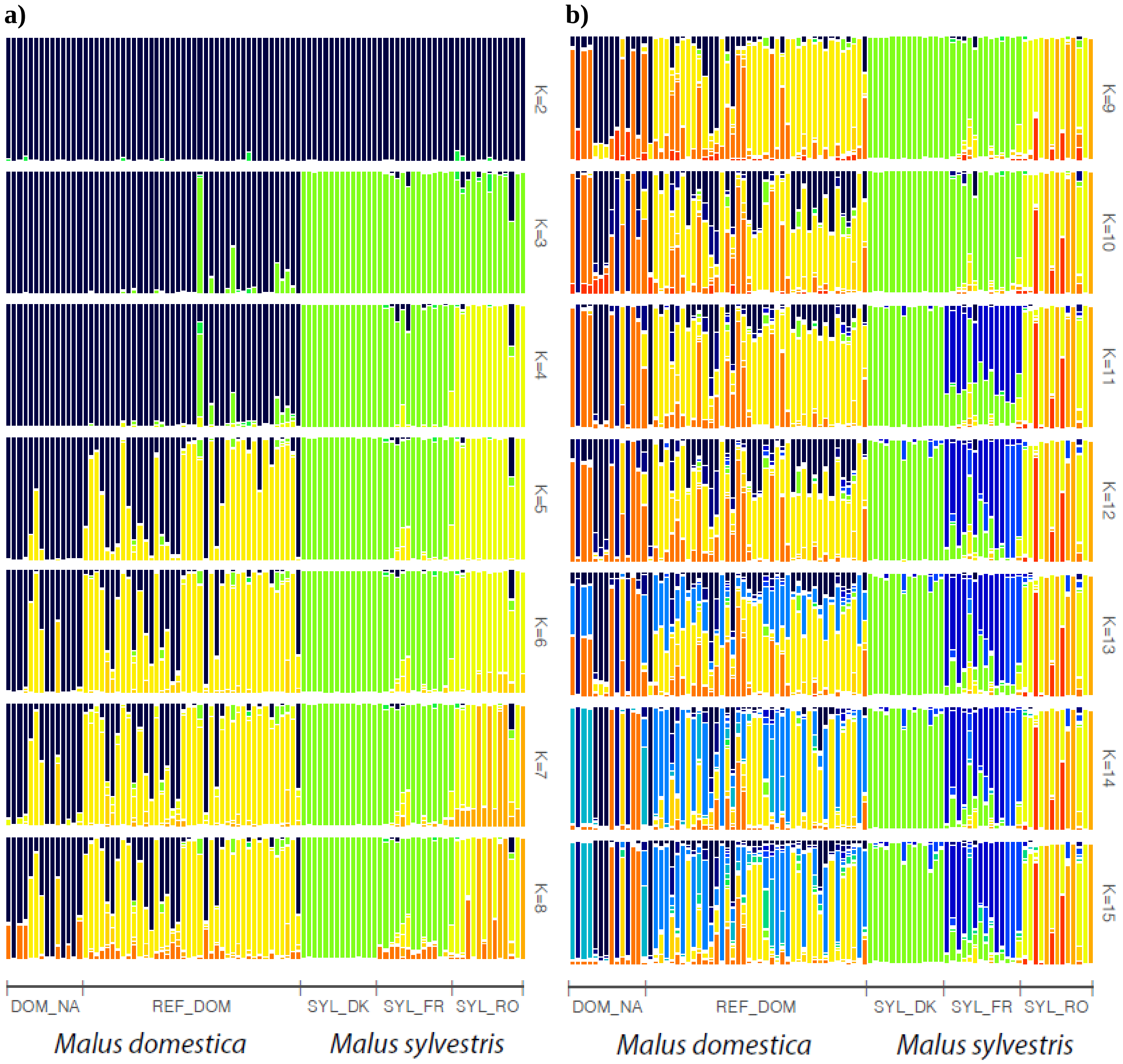

Figure S1. Population structure inferred with STRUCTURE for the Romanian (SYL_RO, *N*=13, one sample could not be genotyped), the French (SYL_FR, *N*=14), the Danish (SYL_DK, *N*=14) *Malus sylvestris* seedlings (green, light blue, yellow, orange colors) and the cultivated *Malus domestica* (dark blue) (DOM_NA, *N* = 14, and, REF_DOM, 40 reference *M. domestica*) for **a)** *K*=2 to *K*=8 and for **b)** *K*=9 to *K*=15.

2. Supplementary tables related to the genetic diversity and genetic differentiation estimates of *Malus domestica* and Danish, French and Romanian *Malus sylvestris* seedlings

Table S1. Genetic diversity estimates for the three *Malus sylvestris* populations (i.e. individuals assigned to the population with a cumulative membership coefficient of >0.9 to the clusters detected for a group of seedlings from the same geographic origin, i.e. DK, FR, RO) detected with STRUCTURE for *K*=14 (SYL_RO, *N*=11, SYL_DK, *N*=12, SYL_FR, *N*=11) and the cultivated *Malus domestica* (DOM_NA, *N* = 14, and, REF_DOM, 40 reference *M. domestica*). *: 0.05<P-value<0.01; **: P-value<0.001

Population	Cluster	*N*	H_O	H_E	F_{IS}
DOM_NA	8 clusters	15	0.737	0.779	0.05
REF_DOM		40	0.813	0.784	-0.04
SYL_DK	1 cluster (green)	12 (2 DK-FR hybrids removed)	0.820	0.801	-0.03
SYL_FR	2 clusters (dark and light blue)	7 (6 samples removed: 3 crop-wild hybrids, and 4 FR-DK wild-wild hybrids)	**0.578**	**0.646**	**0.11****
SYL_RO	3 clusters (orange, red and yellow)	11 (2 samples removed: 1 crop-wild hybrids, and 1 RO-FR wild-wild hybrid)	0.767	0.784	0.02

N=Number of individuals; H_O: observed heterozygosity; H_E: expected heterozygosity, FIS, inbreeding coefficient, bold values: P-values<0.001, cluster: cluster(s) detected and used to cumulate membership coefficients for *K*=14 and sort the crop-to-wild hybrids and wild-wild hybrids, total, *N*=5 individuals were removed from the initial dataset (ID).

Table S2. Pairwise F_{ST} (lower diagonal) and Jost's D (upper diagonal) among the three populations (i.e., cluster inferred with STRUCTURE including plants with a membership coefficient of up to 0.90 for the focal cluster, SYL_RO, *N*=11, SYL_DK, *N*=2, SYL_FR, *N*=7) and the cultivated *Malus domestica* (DOM_NA, *N* = 8, and, REF_DOM, 40 reference *M. domestica*). All values were significant.

	DOM_NA	REF_DOM	SYL_DA	SYL_FR	SYL_RO
DOM_NA		0.18	0.70	0.57	0.60
REF_DOM	0.05		0.68	0.60	0.52
SYL_DA	0.22	0.20		0.22	0.64
SYL_FR	0.14	0.13	0.08		0.50
SYL_RO	0.11	0.09	0.17	0.09	
